# Comparative Analysis of the Effect of Dietary Supplementation with Fermented and Water-Extracted Leaf Extracts of *Eucommia ulmoides* on Egg Production and Egg Nutrition

**DOI:** 10.3390/foods13101521

**Published:** 2024-05-14

**Authors:** Juanhua Yang, Yunfan Wang, Lingyan Zheng, Mijun Peng, Yongzhai Mai, Xuesong Wang

**Affiliations:** 1 Chinese Academy of Inspection & Quarantine Greater Bay Area, Zhongshan 528437, China; yangjh@caiqgba.org.cn (J.Y.); wangyf@caiq.org.cn (Y.W.); zly@caiqgba.org.cn (L.Z.); pengmj@caiqgba.org.cn (M.P.); 2 Scientific Observing and Experimental Station of Fishery Resources and Environment in the Middle and Lower Reaches of Pearl River, Key Laboratory of Prevention and Control for Aquatic Invasive Alien Species, Fishery Ecological Environment Monitoring Center of Pearl River Basin, Ministry of Agriculture and Rural Affairs, Guangdong Provincial Key Laboratory of Aquatic Animal Immunology and Sustainable Aquaculture, Pearl River Fisheries Research Institute, Chinese Academy of Fishery Sciences, Guangzhou 510380, China; 3 Guangdong Provincial Key Laboratory of Chemical Measurement and Emergency Test Technology, Guangdong Provincial Engineering Research Center for Ambient Mass Spectrometry, Institute of Analysis, Guangdong Academy of Sciences (China National Analytical Center, Guangzhou), Guangzhou 510070, China

**Keywords:** *Eucommia ulmoides* leaf, palatability, fermented extract, water extract, hen industry

## Abstract

Although the water extract of *Eucommia ulmoides* leaf (WEE) promotes egg laying in hens, its palatability is poor. To improve the palatability of *E. ulmoides* leaf, probiotic fermentation was used, and fermented extract *E. ulmoides* leaf (FEE) was prepared using *Lactiplantibacillus plantarum*. The safety of FEE was investigated using a long-term toxicity test, and no oxidative damage, inflammatory reactions, or histological lesions were observed in the experimental rats receiving dietary supplementation of FEE at 200 mg/kg, suggesting that FEE is suitable for long-term feeding. Subsequently, dietary supplementation of FEE (group C) in comparison with dietary supplementation of WEE (group B), as well as a control (group A), was applied in the hen industry. Laying performance, egg quality, egg nutrition, egg flavor, and the gut microbiome were analyzed comparatively. Interestingly, the laying rate was observed to be four percentage points higher with dietary supplementation of FEE at 200 mg/kg compared with the control and two percentage points higher compared with the dietary addition of WEE at the same dosage. Simultaneously, a slight upregulation in daily feed consumption was determined in the FEE-supplemented group compared with the blank control and the WEE-supplemented group, indicating that the inclusion of FEE stimulated the hens’ appetite. Moreover, variations in egg amino acids, fatty acids, and volatile components were obtained with either dietary addition, FEE or WEE, implying that dietary supplementation of the fermented and water-extracted *E. ulmoides* leaf extracts contributed to egg flavor change. Furthermore, variations in the gut microbiota were mediated by FEE, increasing the relative abundance of the genus *Lactobacillus*. These alterations in gut microbiota were tightly related to improved laying performance and egg flavor changes. Our results indicate that FEE is a better alternative feed additive in the hen industry than WEE.

## 1. Introduction

Since China has the largest population of any nation, its egg consumption is astonishing. Statistically, domestic egg consumption in China exceeded 30 million tons in 2020, and its output of poultry products ranked first worldwide in the past twenty years [1]. By 2024, the amount of egg production is expected to reach 33 million tons, and the annual output value is expected to exceed CNY 300 billion. To meet this production requirement, antibiotics are commonly used to ensure the normal growth of laying hens due to their anti-disease (e.g., amoxicillin inhibits *Escherichia coli*) and growth-promoting effects [2,3]. However, because several health and environmental problems have been created by antibiotics over time (e.g., chronic toxicity, gene mutation, and antibiotic residue) [4,5,6], their market circulation was expressly prohibited by China in 2020. Therefore, an alternative to antibiotics is urgently needed. Currently, traditional medicine plants in China have been applied in the poultry industry and have huge potential for serving as feed additives [7,8].

As a unique traditional medical plant of China, *Eucommia ulmoides* Oliv. possesses anti-hypertensive, lipid-lowering, and antioxidant advantages [9,10,11]. Currently, numerous natural active components can be characterized from *E. ulmoides* leaf via separation, purification, and identification [12]. Of these, lignans and iridoids are considered key chemotaxonomic markers and exhibit multiple pharmacological activities, e.g., liriodendrin and (+)-syringaresinol of lignans have a satisfactory antihypertension effect, whereas genipin, geniposidic acid, and aucubin of iridoids have a remarkable anti-aging effect [13]. In addition, these iridoids (genipin, geniposidic acid, and aucubin) are considered phytoestrogen compounds [14,15], which can function as modulators of estrogen receptors in animals and stimulate reproduction consequently. Our previous study certified that dietary supplementation of *E. ulmoides* leaf extract at 200 mg/kg (*w*/*w*) could reinforce the laying performance of hens [11]. However, although *E. ulmoides* leaf extract has great potential in the poultry industry, it is bitter, which limits its application due to its poor palatability.

Probiotic fermentation is an effective way to increase the palatability of Chinese medicine plants, altering their flavor and taste [16]. As a simple, cheap, and environmentally friendly method, probiotic fermentation can increase the active components of plant extract via cell wall structure destruction, and it has the advantage of reducing anti-nutritional factors [17]. Simultaneously, the living probiotics colonize the animal intestine and reshape the gut microbiota, forming a competitive advantage in the ecological niche and suppressing the growth of pathogenic bacteria [18,19]. Moreover, a previous study showed that the flavor and taste of plants varied after probiotic fermentation and subsequently stimulated the appetite of animals [20]. Therefore, probiotic fermentation might be an ideal approach to improve the palatability of Chinese medicine plants. However, apart from these advantages, the detrimental effects of probiotic fermentation should also be considered, e.g., the generation of biogenic amines, which cause liver damage, block DNA replication, and disturb biosynthesis [21,22]. Given this point, the safety of the fermentation extract should be explored to ensure the healthy farming of poultry.

In this study, a fermented *E. ulmoides* leaf extract (FEE) was prepared, and its safety was assessed using a long-term toxicity test. Meanwhile, FEE was applied in the healthy farming of laying hens and compared with a water extract of *E. ulmoides* leaf (WEE). Laying performance, egg quality, blood indexes, intestinal histology, and the gut microbiome were analyzed, while dietary supplementation of FEE and WEE was compared at the same dosage in a safety assessment. We hope that dietary supplementation of FEE can further enhance the laying performance compared with WEE. Our result benefits the real application of FEE in the poultry industry.

## 2. Materials and Methods

### 2.1. Fermented and Water-Extracted E. ulmoides Leaf Extract Preparation

For FEE preparation, dry leaves of *E. ulmoides* were collected from Zhangjiajie, China, the famous hometown of *E. ulmoides*. The obtained dry leaves were fermented with probiotic *Lactiplantibacillus plantarum* at a concentration of 1.0 × 10^9^ CFU/mL. The solid–liquid ratio between the *E. ulmoides* leaves and probiotic suspension was 1:1 at 35 °C for 48 h. Subsequently, the fermented extract was dried using an electro-thermostatic blast oven at 50 °C for 24 h, which was finally denoted as FEE. Meanwhile, the WEE used in the present study was the same as our former article [13]. Briefly, dry leaves of *E. ulmoides* were extracted twice with ultrapure water (liquid/solid = 1:10, 80 °C, and 1 h), and the extracted liquid was concentrated to 20 Brix via vacuum rotary evaporation. Next, the spray drying method was used to prepare the final extract (WEE) with the inlet and outlet air temperatures set at 150 °C–170 °C and 90 °C–95 °C, respectively. The active components (total polyphenol, total flavonoid, chlorogenic acid, geniposidic acid, and rutin) in WEE and FEE are listed in Table S1, and their contents in FEE were significantly lower than those in WEE.

### 2.2. Animal Experiments

Two trials of animal experiments were conducted: Trial 1, a safety assessment of FEE; Trial 2, the real application of WEE and FEE.

To perform the safety assessment of FEE (Trial 1), pertinent detail was used while following the standard of the long-term toxicity test (GB15193.13-2015) [13]. Forty 100 g Sprague Dawley rats (half male and half female) were acclimated for 1 week and were divided into two groups using an average: the control (CK), which followed the basal diet; the treatment group (CT), which followed basal diet supplementation of 200 mg/kg FEE. Therefore, 20 rats were allocated to each treatment, and 5 rats in one cage were used as the experimental unit. The FEE dosage in the CT group followed our former study, wherein feeding inclusion of WEE at 200 mg/kg was the optimal dosage in the hen industry [11]. The basal diet was purchased from Jiangsu Xietong Shengwu Co., Ltd. (Nanjing, China). The environment conditions were room temperature (23 ± 1 °C) with a relative humidity of 50% ± 5%. During the entire experimental period (90 days), all rats were allowed free access to water.

For real application (Trial 2), WEE and FEE were employed in the hen industry. The feeding experiment was performed at the Baishi Poultry Farm (Zhongshan, China). Six hundred laying hens, named Jing Fen 1, were acclimated for two months. Subsequently, the acclimated five-month-old laying hens were assigned to three groups using the average: group A, the basal diet; group B, basal diet dietary supplementation of WEE at 200 mg/kg; group C, basal diet dietary supplementation of FEE 200 mg/kg. Therefore, 200 hens were allocated to each treatment, and 4 hens in one cage were used as the experimental unit. The basal diet was provided by Zhengda Kangdi Co., Ltd. (Shenzhen, China), and its composition is shown in Table S2. During the entire feeding period (4 months), all hens were allowed free access to water, and the room temperature was always kept at 24 ± 1 °C. An illumination cycle was set at the same time, and the cycling program was 16 h light and 8 h dark. Simultaneously, the laying performance-related parameters were recorded daily, e.g., the number of eggs, number of broken eggs, egg weight, number of hen deaths, and feed consumption.

### 2.3. Sample Collection

In Trial 1, the blood samples (*n* = 20, including both male and female) were sampled from the abdominal aortic vein of rats when they had finished feeding, while, in Trial 2, blood samples (*n* = 10) were sampled from the wing vein of hens when they had finished feeding. Subsequently, the obtained blood samples were immediately centrifuged. The centrifugation time for rats was 15 min with a centrifugal force of 1200 g. In contrast, the centrifugation time for hens was 10 min with a centrifugal force of 1500 g. After centrifugation, the obtained blood samples were used for blood index determination. Meanwhile, six organs (including brain, heart, liver, kidney, spleen, and testes) from rats (*n* = 20) and two organs (liver and spleen) from hens (*n* = 10) were harvested and weighed individually. Meanwhile, five tissues (liver, kidney, stomach, spleen, and pancreas) from rats (*n* = 20) and three intestinal tissues (duodenum, ileum, and jejunum) from hens (*n* = 10) from each group were stored at 4% paraformaldehyde for histopathological examination. Moreover, the cecum contents of hens (*n* = 10) were sampled and preserved for gut microbiome sequencing and analysis. The storage temperature of the obtained blood and tissue samples was −80 °C.

### 2.4. Hematological Analysis

Different hematological analyses were conducted in the two trials. For the safety assessment (Trial 1, *n* = 20), blood routine indexes (including white blood cell (WBC), neutrophil (Neu), lymphocyte (Lym), monocyte (Mon), eosinophil (Eos), basophil (Bas), red blood cell (RBC), hemoglobin (HGB), hematocrit value (HCT), mean corpuscular volume (MCV), mean corpuscular hemoglobin (MCH), mean corpuscular hemoglobin concentration (MCHC), red blood cell distribution width coefficient of variation (RDW-CV), red blood cell distribution width standard deviation (RDW-SD), platelet (PLT), mean platelet volume (MPV), platelet distribution width (PDW), and thrombocytocrit (PCT)); blood biochemical indexes (including albumin (ALB), alkaliphosphatase (ALP), alanine aminotransferase (ALT), aspartate aminotransferase (AST), creatinine (CREA-S), glucose (Glu-G), total cholesterol (TC), triglyceride (TG), total phosphorus (TP), UREA, and *γ*-glutamyl transpeptidase (*γ*-GT)); blood antioxidant indexes (including superoxide dismutase (SOD), glutathione (GSH), molondialdehyde (MDA), and catalase (CAT)); and blood inflammatory factors (including tumor necrosis factor-*α* (TNF-*α*), interleukin-1*β* (IL-1*β*), and interleukin-6 (IL-6)) were detected at GuangZhou KYDbio Technology CO., LTD (Guangzhou, China). The detection of blood routine and blood biochemical indexes was performed using an Animal five-class blood cell analyzer (BC-5000VET) and Animal automatic biochemical analyzer (BS-240VET), respectively (Mindray, Shenzhen, China). The reagents used for blood routine indexes test were Hemolytic agent for veterinary blood cell analysis (V-52LH) and V-P probe cleaning solution (Mindray, Shenzhen, China). The kits used for blood biochemical indexes test were the Albumin kit (Bromcresol Green Method, P/N:105-020590-00), Mindray alkaline phosphatase (ALP) assay kit (AMP buffer Method, P/N:105-020578), Alanine Aminotransferase Kit (lFCC Method, P/N:105-020579-00), Aspartate Aminotransferase Kit (lFCC Method, P/N:105-020580-00), Creatinine Kit (Sarcosine Oxidase Method, P/N:105-020587-00), Glucose Kit (GOD-POD Method, P/N:105-020586-00), Mindray total cholesterol (TC) determination kit (Oxidase Method, P/N:105-020585-00), Mindray Triglyceride (TG) Assay Kit (Oxidase Method, P/N:105-020584-00), Inorganic phosphorus (P) determination kit (phosphomolybdic acid method, P/N:105-020593-00, Urea Kit (Urease-GLDH, UV Method, P/N:105-020583-00), and Gamma-Glutamyltransferase Kit (Szasz Method/lFCC stand, P/N:105-020582-00) (Mindray, Shenzhen, China). Blood antioxidant indexes were assayed by using Beijing Solarbio^®^ colorimetric test kits (Solarbio Life Science Co., Ltd., Beijing, China), while blood inflammatory factors were determined by using Shanghai Enzyme-linked^®^ colorimetric kits (mlbio, Shanghai, China).

For the real application (Trial 2, *n* = 10), three blood immunoglobulins (Ig A, Ig G, and Ig M) were monitored to reflect the immunity response in hens, while the levels of two blood interleukins (IL-1 and IL-6), as well as the blood TNF-*α*, were determined to reflect the inflammatory reaction in hens. All of these tests were conducted using Beijing Solarbio^®^ colorimetric test kits (Solarbio Life Science Co., Ltd., Beijing, China). Meanwhile, blood low-/high-density lipoprotein cholesterol (LDL-C/HDL-C) were detected using the LDL-C kit (DL18A2) and HDL-C kit (DL18A1) (Donglin Bio., Guangzhou, China). Additionally, blood TC and blood TG were also detected to reflect the blood lipid level in hens, while the inclusion of enzymes ALT, AST, ALB, and ALP was evaluated to reflect the liver function of hens. Moreover, blood uric acid (UA), blood urea nitrogen (BUN), and UREA were assessed to reflect the nitrogen metabolism in hens, accompanied by the determination of the blood Ca and blood P, which are involved in the shell strength. All of these indexes in Trial 2 were performed on an Automatic biochemical analyzer (Hitachi 7180) at Rayto Biotechnology Ltd. (Guangzhou, China) using the above-mentioned kits.

### 2.5. Histopathological Examining

For histopathological examination, the liver, kidney, stomach, spleen, and pancreas of rats from Trial 1 (*n* = 20) and the duodenum, ileum, and jejunum of hens from Trial 2 (*n* = 10) were selected and studied. First, these tissues were dehydrated with graded alcohol and xylene; next, these tissues were embedded in paraffin and cooled down at room temperature; third, 4 μm sections were cut from the solid paraffin and then stained with a mixture inclusion of hematoxylin and eosin; lastly, the histological observation of these selected tissues was performed on an automatic image analyzer, which was purchased from ECHO (Chicago, IL, USA). Meanwhile, ImageJ 1.54h (National Institutes of Health, USA), which is free to the public (http://imagej.org, accessed on 15 December 2023), was used for image analysis. Additionally, six well-oriented villus heights and their corresponding crypt depths in the intestinal tissues were measured using the automatic image analyzer.

### 2.6. Laying Performance and Egg Quality Determination

The initial and final body weights of the hens (*n* = 200) were recorded at the beginning and the end of the experiment. Meanwhile, the total number of eggs in each group was added up, and the mean egg weight was calculated. The laying rate and broken egg rate (BER) were assessed as follows: laying rate = total number of eggs/(120*200) × 100%; BER = number of broken eggs/total number of eggs × 100%. In addition, to calculate daily feed consumption, a certain portion of the diet (22,000 g) was added daily, and the rest of the diet was collected. Therefore, the daily feed consumption (DFC) of the hens and the feed conversion rate (FCR) could be calculated. Here, DFC = total amount of feed consumed per day/200; FCR = total feed consumption/total egg weight.

Apart from laying performance, egg quality was evaluated. Twenty eggs (*n* = 20) were randomly selected from each group. An electronic digital caliper (LR44, China) and an electronic scale were applied to measure the egg shape index, eggshell thickness, and shell weight. Further, eggshell strength was detected by Shengtai Ltd. using a tester (Jinan, China). Additionally, determination of the yolk color and Haugh units were performed on an egg analyzer (EA-01, ORKA, Ramat Hasharon, Israel), and the moisture contents of yolk and albumen were detected in an oven under 100 °C for 4 h based on GB 5009.3-2016 [11].

### 2.7. Egg Nutritional Component Analysis

Since amino acids in albumen and fatty acids in the yolk are the main nutritional components of eggs, their contents were detected. For albumen amino acids detection (*n* = 3), GB 5009.124 was selected [11]. Briefly, the weighed solution was hydrolyzed using a 6 M HCl solution at 110 °C for 22 h. After cooling down, the solution was filtered with a cellulose ester membrane (pore size 0.22 µm) and analyzed using an L-8900 Amino Acid Analyzer (Hitachi, Kyoto, Japan) equipped with a Hitachi custom ion exchange resin and electronic heating reaction unit. The injection volume was 20 μL. The contents of amino acids were determined to be 570 nm and 440 nm within 60 min. For yolk fatty acid detection (*n* = 3), GB 5009.168 was applied [11]. Briefly, the weighed sample was hydrolyzed using an 8.3 M HCl solution. Then, the fat was extracted using a mixture solution comprising petroleum ether and ether (*v*/*v*, 1:1) and dried under nitrogen blowing. Subsequently, the fat extract was saponified by adding 2% sodium hydroxide dissolved in methanol. Meanwhile, fatty acid methyl esters (FAMEs) were prepared via transesterification with a 14% boron trifluoride methanol solution (Macklin, Shanghai, China) and dissolved in 5 mL of hexane. Gas chromatographic analysis of FAMEs was performed on a GC-2010 plus (Shimadzu, Kyoto, Japan) equipped with an HP-88 fused silica capillary column (100 m × 0.25 mm internal diameter, 0.2 μm film thickness, Agilent, Santa Clara, CA, USA) and a flame ionization detector. The temperature program for separation was as follows: first, 130 °C for 1 min; second, increased from 130 °C to 180 °C at a rate of 10 °C/min; third, increased from 180 °C to 215 °C at a rate of 2.75 °C/min; fourth, increased from 215 °C to 235 °C at a rate of 5 °C/min; last, maintained at 235 °C for 10 min. The split ratio was set at 5:1, and helium was designated as the carrier gas with a flow rate of 1.2 mL/min. Additionally, the cholesterol content in the yolk (*n* = 3) was analyzed by following the aforementioned method, which we applied previously [11].

### 2.8. Safety Assessment of Eggs

Fresh eggs (*n* = 3) were used for the safety assessment. Four microbial indices (including Salmonella, aerobic plate count, molds, and coliforms) in eggs were monitored by following the GB 4789.4-2016, GB 4789.2-2016, GB 4789.15-2016, and GB 4789.3-2016 standards [11], respectively. Meanwhile, aflatoxin B1 was detected by following the GB 5009.22-2016 standard [11], while Pb and Cd (typical heavy metals) were detected by following the GB 5009.74-2014 standard [11].

### 2.9. Validation of the Volatile Components of Eggs

The headspace–gas chromatography–ion mobility spectrometry (HS-GC-IMS) analysis of egg samples (*n* = 5) was conducted at Hanon Scientific Instrument Co., Ltd. (Beijing, China) [11,12]. During determination, a gas chromatography–ion mobility spectrometry (GC-IMS) equipped with an autosampler, and headspace sampling unit was selected, provided by Agilent (Dortmund, Germany). Briefly, the egg sample (2 g) was transferred into a glass vial (20 mL), which was sealed with a silicon septum and magnetic metal crimp. Simultaneously, 1000 mL of solution was injected via a heated syringe at 95 °C. GC-IMS analysis was performed on an Agilent 490 micro gas chromatograph equipped with an MXT-5 (15 m × 0.53 mm) column. The carrier gas program was 2 mL/min from 0 to 2 min and amplified up to 100 mL/min over 18 min. Drift gas with a 150 mL/min rate was also needed. The obtained data were examined using principal component analysis (PCA) and partial least squares discriminant analysis (OPLS-DA). Meanwhile, a gallery plot of volatile components was constructed to exhibit the differences between the blank control and the WEE/FEE-supplemented groups.

### 2.10. Gut Microbiome Sequencing and Analysis

The gut microbiota was applied to 16S rRNA gene amplicon sequencing. The DNA sample of cecum contents (*n* = 3) was extracted using the relative DNA isolation kits (Qiagen NV, Düsseldorf, Germany). A typical forward primer paired with a typical reverse primer was used to amplify the specific regions of the obtained DNA, and the sequences of these two primers were as follows: 341F, 5′-CCTACGGGNGGCWGCAG-3′, and 805R, 5′-GACTACHVGGGTATCTAATCC-3′. During amplification, the thermocycling program was set as follows: denaturation at 98 °C for 30 s; amplification with 35 cycles at 98 °C for 10 s, 54 °C for 30 s, and 72 °C for 45 s; and elongation at 72 °C for 10 min. After amplification, the obtained amplicons were purified and quantified first, and the V3–V4 region was applied to a 16S rRNA sequencing on an Illumina HiSeq 2500 platform (Illumina, San Diego, CA, USA). For clean data collection, denoising was necessary, and a divisive amplicon denoising algorithm (DADA2) was carried out. Subsequently, amplicon sequence variants (ASVs) with 100% similarity were clustered, and the reference ASVs were further matched with the SILVA database. The *α*-diversity of bacterial community among the three tested groups was detected by calculating the Chao 1 and Shannon indexes. In contrast, the *β*-diversity of the bacterial community was detected based on the Bray–Curtis distances, which were coupled with principal coordinate analysis (PCoA). Meanwhile, permutational multivariate analysis of variance (PERMANOVA) was conducted to validate the *β*-diversity. Based on criteria of *r* ≥ 0.7 and *p* < 0.05, potential keystone species were screened out based on co-occurrence networks. Additionally, a random forest test (RFT) was carried out to identify the potential keystone species, collated by means of ten-fold cross-validation. In the RFT, groups B and C were designated as the “NOT” group compared with group A. Moreover, to identify imputed microbial functions, phylogenetic investigation of communities by reconstruction of unobserved states (PICRUSt2) was used. Furthermore, at the threshold of *r* ≥ 0.3 and *p* < 0.05, the multiple correlations between keystone species, imputed functional profiles, and nutritional components were constructed and finally visualized as a Sankey plot (RStudio software, version 2022.07.0).

### 2.11. Statistical Analyses

In the present study, the mean ± standard deviation (SD) was applied to express the obtained data. Meanwhile, one-way analysis of variance (one-way ANOVA) analysis (SPSS 19.0) was used to examine the significant difference between the blank control and the WEE/FEE-supplemented groups, with *p* < 0.05 considered to indicate statistical significance. Moreover, multiple comparisons were collated via a Tukey test. For figure and table preparation, either WPS Excel or GraphPad Prism (San Diego, CA, USA) was used.

## 3. Results

### 3.1. Safety Assessment of FEE

During the entire long-term toxicity test, no rat mortality was observed. Interestingly, a body weight elevation was observed in rats in the CT group in comparison with the CK group (Table S3), especially for male rats, with a significant difference between the two groups (*p* < 0.05). Meanwhile, the brain, heart, and kidney weights of male rats also increased in the CT group when compared with the CK group (*p* < 0.05), suggesting that dietary supplementation of FEE was growth promoting. Additionally, histopathological examination of the five selected tissues (liver, spleen, kidney, stomach, and pancreas) revealed no pathological changes (e.g., cytoplasmic vacuolation, inflammatory infiltration, cell gap increasing, and abnormal cellular change) between CK and CT groups, manifesting that long-term supplementation FEE did not cause histology lesion (Figure S1). Furthermore, routine blood indexes (Table S4), blood biochemistry indexes (Table S5), antioxidant indicators (SOD, GSH, MDA, and CAT), and inflammatory factors (TNF-*α*, IL-1*β*, and IL-6) were at the same level between CK and CT groups (Table S6), implying that the dietary addition of FEE did not induce oxidative damage and an inflammatory reaction in rats. These results showed the prepared FEE was safe and could be used for healthy farming.

### 3.2. Laying and Growth Performance of Hens

During the entire experiment, no mortality of hens was determined. Remarkably, the laying rate was the highest in group C (93.44%), followed by B (91.24%), and was the lowest in group A (89.23%), indicating that the dietary addition of FEE contributed to laying performance enhancement further in comparison with the dietary supplementation of WEE (Table 1). Meanwhile, a lower BER in groups B (0.08%) and C (0.09%) was found in comparison with that in group A (0.12%), implying that feeding inclusion of WEE and FEE benefited egg production. In contrast, the mean egg weight and daily feed consumption (DFC) revealed no significant difference among the three tested groups (*p* > 0.05); regardless, the mean egg weight in group B (64.44 g) was two grams heavier than that in groups A (62.43 g) and C (62.45 g) and the daily feed consumption (DFC) in group C (106.18 g) was slightly higher than that in the other two groups (group A, 103.08 g; group B, 103.66 g). Based on these alterations, the FCR showed no apparent difference among the three tested groups. In addition, the final body weight of hens exhibited no significant difference between the FEE/WEE-added groups and the blank control (*p* > 0.05), which was in line with the results, wherein the organ coefficients of liver and spleen showed no difference among the three tested groups, implying that daily feeding with WEE and FEE did not affect the growth performance of hens.

### 3.3. Quality of Eggs

Remarkably, the values of egg-quality-related indexes in the blank control (group A) were equivalent to these in WEE/FEE-supplemented groups (groups B and C) (Table S7), e.g., the yolk color in group A was 6.60, which was similar to that in groups B (6.56) and C (6.48), suggesting that the egg quality remained the same in groups with FEE and WEE added in comparison with the blank control. Therefore, dietary supplementation with WEE and FEE did not affect these egg-quality-related indexes.

### 3.4. Albumen Amino Acid and Yolk Fatty Acid

Regarding albumen amino acids, the proportion of seven essential amino acids (EAA) in group A (41.43%) was close to that in groups B (41.06%) and C (40.74%) (Table 2). Additionally, Asp, Glu, Lys, Ser, and Leu were the top five amino acids present in albumen as their contents were greater than 1.00 g/100 g FW (fresh weight), especially Glu. Meanwhile, the delicious amino acid (DAA) proportions surpassed 43% in the three test groups, higher than the standard of 40%, implying that the eggs were of high quality. Variations in bitter amino acid (BAA) were observed, wherein the content of Arg increased in the FEE-supplemented group (group C) compared with the other two groups, while the content of His decreased in both WEE- and FEE-supplemented groups (groups B and C). Regarding yolk fatty acid, the total content in group A (33.46 g/100 g FW) was similar to that in groups B (33.52 g/100 g FW) and C (33.73 g/100 g FW) (Table 3). In addition, C16:0, C16:1n7, C18:0, C18:1n9c, and C18:2n6c were the top five fatty acids, as their contents exceeded 1.00 g/100 g FW, especially C16:0 and C18:1n9c. Variations in yolk fatty acid were also determined, wherein the contents of C16:1n7, C18:2n6c, and C18:3n3 descended in both WEE- and FEE-supplemented groups (groups B and C) when compared with the blank control (group A), whereas the content of C18:0 and C18:1n9c elevated in these two groups in comparison with the blank control (group A). These results showed that WEE and FEE dietary supplementation shared similar profiles in terms of egg nutrient changes.

### 3.5. Egg Volatile Components

**Figure 1:**
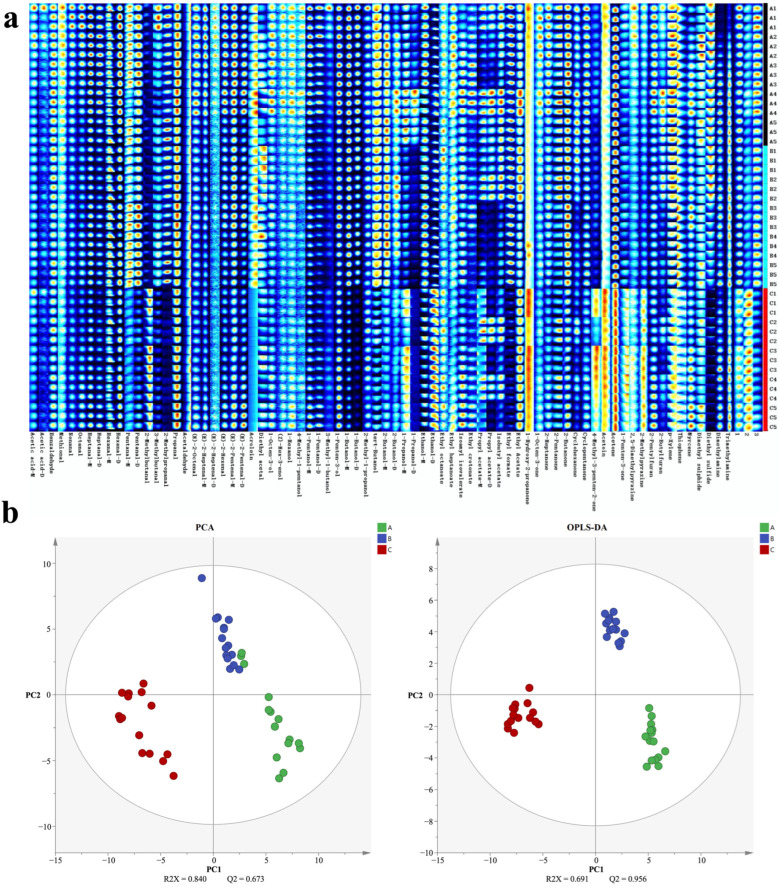
Effects of feeding inclusion fermented and water extracted leaf extracts of *E. ulmoides* on volatile components of eggs. (**a**) Differences in volatile components exhibiting as the gallery plot according to HS-GC-IMS. (**b**) Differences in volatile components exhibiting as the PCA and the OPLS-DA plots among the three tested groups. Abbreviations: A group, control; B group, dietary supplementation water extracted leaf extract of *E. ulmoides*; C group, dietary supplementation fermented leaf extract of *E. ulmoides*.

Seventy-four volatile components were identified in the eggs (Table S8). Based on the gallery plot, the main volatile components were classified into acids, alcohols, aldehydes, ketones, and esters (Figure 1a). Of these, the profiles of volatile components among the three tested groups showed an apparent difference, especially for group C, which exhibited significant differences compared with the other two groups (groups A and B). Simultaneously, volatile components were clearly separated between the blank control (group A) and the WEE/FEE-supplemented groups (groups B and C) in the PCA and the OPLS-DA plots (Figure 1b), indicating that dietary supplementation with FEE could change the flavor of eggs in comparison with the WEE-added group, as well as the blank control.

### 3.6. Safety of Eggs

No Salmonella was detected in the eggs among the three tested groups, indicating that none of this typical pathogenic bacterium was introduced with the daily addition of FEE. Simultaneously, the aerobic plate count, molds, and coliforms in eggs among the three tested groups were all lower than 100, 3, and 10 CFU/mL (Table S9), respectively. Further, aflatoxin B1 and heavy metals (Pb and Cd) were not detected in the eggs. All of these indices met the national food safety standards of China.

### 3.7. Blood Biochemical Parameters of Hens

The content of ALP content significantly decreased with the dietary supplementation of WEE (group B) and FEE (group C) in comparison with the blank control (group A) (*p* < 0.05). At the same time, no significant difference was determined in the contents of ALT, AST, and ALB among the three tested groups (*p* > 0.05, Table 4). Significant declines in UREA, UA, Ca, and P contents were observed with the dietary supplementation of FEE (group C) in comparison with the blank control (group A) (*p* < 0.05). In contrast, only the contents of UA and Ca significantly decreased in the WEE-added group (group B) compared with the blank control (group A). Moreover, the contents of inflammatory factors (TNF-*α*, IL-1, and IL-6) also revealed no significant difference among the three tested groups (*p* > 0.05). Therefore, dietary supplementation with FEE can affect liver function, purine metabolism, and shell-strength-related parameters, similarly to the dietary addition of WEE.

### 3.8. Lipid and Immunoglobulin Indexes of Hens

**Figure 2:**
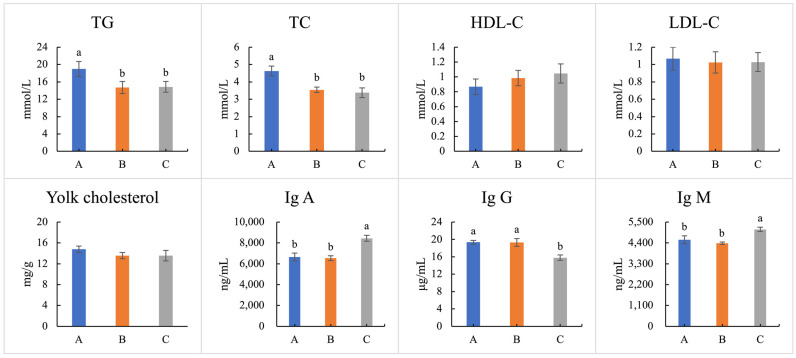
Effects of feeding inclusion fermented and water extracted leaf extracts of *E. ulmoides* on lipid and immunoglobulin indexes. Different small letters indicate significant differences at *p* < 0.05 level under different treatments. Abbreviations: A group, control; B group, dietary supplementation water extracted leaf extract of *E. ulmoides*; C group, dietary supplementation fermented leaf extract of *E. ulmoides*; TG, triglyceride; TC, total cholesterol; HDL-C, high-density lipoprotein cholesterol; LDL-C, low-density lipoprotein cholesterol; Ig A, immunoglobulins A; Ig G, immunoglobulins G; Ig M, immunoglobulins M.

Regarding lipid indices, although the contents of blood HDL-C, blood LDL-C, and yolk cholesterol revealed no significant differences among the three tested groups (*p* > 0.05), the blood TG and blood TC contents in the WEE/FEE-supplemented groups (groups B and C) significantly decreased in comparison with the blank control (group A) (*p* < 0.05, Figure 2), indicating that feeding inclusion of WEE or FEE had lipid-lowering benefits. Regarding immunoglobulin indexes, the contents of Ig A and Ig M in the control (group A) and the WEE-supplemented group (group B) were significantly lower than those in the FEE-supplemented group (group C) (*p* < 0.05). In contrast, the content of Ig G in the control and WEE-supplemented groups (groups A and B) was significantly higher than that in the FEE-supplemented group (group C), suggesting that daily supplementation of FEE potentially participated in immunoregulation when compared with daily supplementation of WEE.

### 3.9. Intestinal Histology of Hens

**Figure 3:**
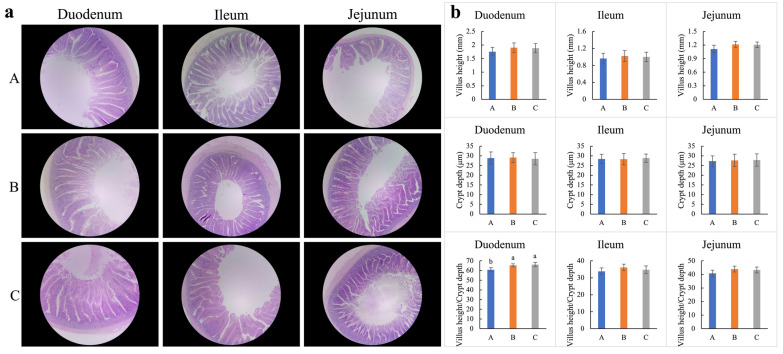
Effect on intestinal histology as feeding inclusion fermented and water extracted leaf extracts of *E. ulmoides*. (**a**) Real photomicrographs of duodenum, ileum, and jejunum (40×). (**b**) The height of villus, depth of crypt, and the ratio between villus and crypt in duodenum, ileum, and jejunum. Different small letters indicate significant differences at *p* < 0.05 level under different treatments. Abbreviations: A group, control; B group, dietary supplementation water extracted leaf extract of *E. ulmoides*; C group, dietary supplementation fermented leaf extract of *E. ulmoides*.

Although a slight elevation in villus heights of the duodenum, ileum, and jejunum were observed with the dietary supplementation of WEE (group B) and FEE (group C) when compared with the blank control (group A), no significant difference was determined (*p* > 0.05) (Figure 3a,b). Additionally, the crypt depth of the duodenum, ileum, and jejunum in groups B and C remained the same as those in group A without a significant difference (*p* > 0.05). Since the villus height increased, the ratio of the villus height to the crypt depth in the duodenum was therefore enlarged in the WEE- and FEE-supplemented groups (groups B and C) in comparison with the blank control (group A), with a significant difference (*p* < 0.05). Therefore, dietary supplementation of WEE and FEE potentially contributes to intestinal histology melioration.

### 3.10. Gut Microbiome

**Figure 4:**
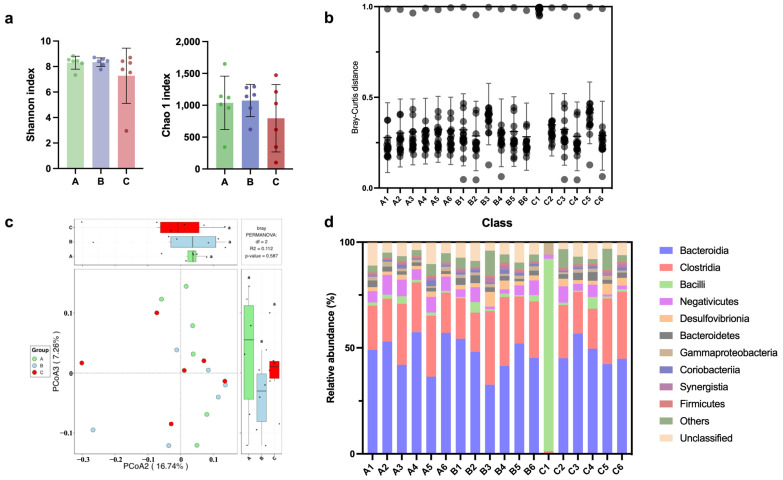
Dynamics of gut microbiota. (**a**) The *α*-diversity of gut microbiota. (**b**) The Bray–Curtis distance across individual microbiota. (**c**) The principal coordinate analysis (PCoA) of gut microbiota. (**d**) The relative abundance of bacterial at the class level. Abbreviations: A group, control; B group, dietary supplementation water extracted leaf extract of *E. ulmoides*; C group, dietary supplementation fermented leaf extract of *E. ulmoides*. A1–6, B1–6, and C1–6 represent the serial number of samples in 16S rRNA gene amplicon sequencing of the three tested groups.

Although the *α*-diversity (Shannon and Chao 1) of the gut microbiota slightly decreased with the dietary addition of FEE (group C), no significant difference was determined among the three tested groups (Figure 4a). Except for one sample of group C (Sample C1), the Bray–Curtis dissimilarity and the relative abundance of bacterial were the same in terms of the class level between the blank control (group A) and the WEE/FEE-supplemented groups (groups B and C) (Figure 4b,d), wherein *Bacteroidia*, *Clostridia*, and *Bacilli* remained the dominant bacterial class. Additionally, no obvious deviation in gut microbiota was found among the three tested groups based on principal coordinate analyses (PCoAs) (Figure 4c).

**Figure 5:**
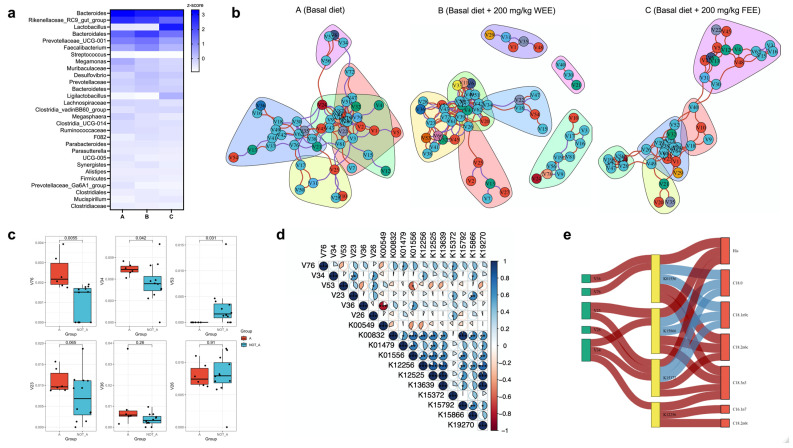
Taxonomy and function composition of gut microbiota. (**a**) Heatmap of the top 30 gut microbiota at the genera level. (**b**) Co-occurrence network of the significantly different genera. (**c**) Keystone species according to RFT (asterisk indicate statistically significant differences). (**d**) Correlation between the keystone species and the predicted functional profiles of gut microbiota. (**e**) Sankey plot of the relationship between gut microbiota and eggs nutritional components. The keystone species are the green rectangles, the imputed functional profiles of gut microbiota are the yellow rectangles, and the nutritional components are the red rectangles. Lines in red represent the positive relationship between categories, while lines in blue represent the negative relationship. Abbreviations: A group, control; B group, dietary supplementation water extracted leaf extract of *E. ulmoides*; C group, dietary supplementation fermented leaf extract of *E. ulmoides*; WEE, water extracted leaf extract of *E. ulmoides*; FEE, fermented leaf extract of *E. ulmoides*.

The top 30 bacteria at the genera level were screened out, and *Lactobacillus* probiotics were enriched in the FEE-supplemented group (group C) in comparison with the other two groups (groups A and B) (Figure 5a), indicating that the fermentation probiotics used dominated the ecological niche in the intestinal microecology. Meanwhile, the edges (168) and transitivity (0.623) were the highest in group B based on co-occurrence network analysis (Figure 5b), indicating that dietary addition of WEE benefited gut microbiota regulation. Six keystone species were obtained according to RFT, including *Romboutsia* (V76), *Subdoligranulum* (V34), *Helicobacter* (V53), *Frankiales* (V36), UCG-005 (V23), and unclassified *Firmicutes* (V26). Among them, the relative abundance of two pathogenic bacteria, *Romboutsia* and *Subdoligranulum*, significantly decreased with the dietary supplementation WEE/FEE (Figure 5c). In addition, *Subdoligranulum* exhibited significant positive correlations with amino acid metabolism enzymes (K12256, putrescine—pyruvate transaminase; K15372, taurine—2-oxoglutarate transaminase; K15866, 2-(1,2-epoxy-1,2-dihydrophenyl)acetyl-CoA isomerase) (Figure 5d). Further, the Sankey plot revealed that the abundance of *Frankiales*, *Romboutsia*, and UCG-005 (V23) reduced with the dietary supplementation of WEE/FEE and participated in amino acid metabolism (K01556, kynureninase; K15866; K15372), which was tightly involvement with His alteration (Figure 5e). Moreover, decreases in keystone species (V36, V76, V23, and V34) were strongly associated with K01556, K15866, and K15372 enzymes and facilitated fatty acid (C18:0 and C18:1n9c) elevation in eggs with the daily supplementation of WEE/FEE.

## 4. Discussion

### 4.1. The Prepared Fermented E. ulmoides Leaf Extract Is Safe for Long-Term Feeding

To assess the in vivo safety of the prepared fermented *E. ulmoides* leaf extract (FEE), a long-term toxicity test was necessary [23]. Such a test can demonstrate whether daily feeding with FEE under long-term administration will cause tissue and organ damage and can also ensure the feeding dose in real applications. Based on the stipulated index of the long-term toxicity test, the growth performance, blood indexes, and histomorphology of rats were analyzed, and the dosage of FEE (200 mg/kg) was set in line with our former research, wherein dietary supplementation of WEE at 200 mg/kg contributed to laying performance elevation [11]. In the present study, no rat mortality was observed during the whole experiment. The body weights of the rats increased with the dietary addition of FEE in comparison with the blank control, which was in accordance with the result that feeding inclusion of WEE facilitated the growth performance of rats [13]. Additionally, the blood indexes (blood routine and blood biochemistry), the antioxidant indexes, and the inflammatory factors were at the same level in the FEE-supplemented group and the blank control, implying that daily feeding with FEE was safe for rats since no in vivo oxidative damage or inflammatory reaction was induced. Meanwhile, no tissue (liver, spleen, kidney, stomach, pancreas) lesions were observed in rats in the blank control and the FEE-supplemented groups, implying that dietary addition of FEE did not induce tissue damage. Based on the results of the long-term toxicity test, we believe that dietary supplementation of FEE is safe for real application in the hen industry. Consistently, the body health of hens improved with feeding supplementation of FEE in real applications. Regardless of no significant difference being detected for LDL-C and HDL-C among the three tested groups, the blood TG and TC contents of hens were downregulated with daily feeding of FEE in comparison with the blank control, suggesting that dietary supplementation of FEE was conducive to blood lipid lowering. This phenomenon was similar to the dietary supplementation WEE in the hen industry. Numerous studies have reported that *E. ulmoides* can ameliorate hypertriglyceridemia via up-regulating hepatic *α*-, *β*-, and *ω*-oxidation-related genes in rats [24] and further participate in blood lipid metabolism via regulating the expression of PPAR*γ* [25,26]. Therefore, FEE might share the same mechanism with *E. ulmoides* in lowering blood lipids. Moreover, FEE further showed its UA-lowering ability, and a significant decrease in UA content was observed with the feeding inclusion of FEE, which was also similar to the dietary supplementation of WEE. These results were consistent with the finding in hyperuricemic mice that *E. ulmoides* had anti-hyperuricemia advantages [27]. Everything considered, we thus affirm that the prepared FEE is safe for long-term feeding.

### 4.2. Feeding Inclusion FEE Benefits in Laying Performance Elevation

Inconsistent with the enhancement in the growth performance of rats, dietary supplementation of FEE did not facilitate the growth performance of hens. Our previous work had certified that the laying performance of hens was elevated with the dietary supplementation of WEE at 200 mg/kg [11]; in the present study, two more percentage point elevations in laying performance were determined with the daily feeding of FEE in comparison with daily feeding of WEE. Therefore, dietary supplementation of FEE further contributed to enhancing laying performance. In addition, the egg quality was analyzed between the blank control and the WEE/FEE-supplemented groups. It revealed no significant difference, suggesting that dietary supplementation of FEE could maintain the normal quality of eggs along with laying performance upregulation. Our former work illustrated that the improved laying performance triggered by dietary supplementation of WEE might be ascribed to phytoestrogen iridoids in *E. ulmoides* [11,14], such as aucubin and geniposidic acid, which could serve as estrogen receptor modulators and thus improve egg production. Interestingly, these components were also found in FEE, despite their contents in FEE all being significantly lower than those in WEE. Therefore, FEE potentially shares the same mechanism for stimulating egg production based on these same components. Further, due to egg production being associated with nutrient absorption in the intestine, the histomorphology of the intestine was analyzed. Studies in the literature have shown that a higher villus height and a greater villus height/crypt depth ratio facilitate nutrient absorption [28,29]. Consistently, slight elevations in the villus height and villus height/crypt depth ratio were detected with the dietary addition of WEE and FEE when compared with the blank control, but no difference was found between the WEE- and FEE-added groups, implying egg production’s further elevation in the FEE-supplemented group might be ascribed to other factors. Previous evidence has pointed out that the gut microbiota is closely involved in nutrient absorption [30]. In the present research, probiotic fermentation was applied to improve the palatability of *E. ulmoides* leaf, and we found that the gut microbiota varied at the genera level with dietary supplementation of FEE, exhibiting that the genera of fermentation probiotics (*Lactobacillus*) were enriched in the FEE-supplemented group compared with the other two groups. Additionally, former studies have reported that a fermented extract can ameliorate the appetite of animals [31] and increase feed consumption [32]. Remarkably, regardless of no significant difference being detected, the daily feed consumption of hens increased with the dietary addition of FEE, which was slightly higher than that for the dietary addition of WEE and the blank control. Therefore, the mechanism wherein the dietary supplementation of FEE benefited the laying rate might result from feed consumption increase, which was related to the alteration of gut microbiota. Combining the above-mentioned aspects, we can thus summarize that the increase in egg production can be ascribed to the phytoestrogen role of active components in FEE and the feed-attracting role of FEE.

### 4.3. Variations in Egg Nutrition Is Mediated by Gut Microbiota with Feeding Inclusion of FEE

Albumen amino acid and yolk fatty acid are the major nutritional components of eggs [33]. Recent research has shown that dietary supplementation of *E. ulmoides* could induce amino acid and fatty acid variation via gut bacterial mediation [12]. For instance, amino acid metabolism was mediated by a beneficial bacterium [34] and could alter the composition of amino acids in eggs [11]. In the present work, variations in bitter amino acids were observed in the FEE-supplemented group compared with the blank control, e.g., a His decrease and Arg increase. However, only a His decrease was determined in the WEE-supplemented group in comparison with the blank control. Similarly, the fatty acids varied, e.g., the contents of C16:1n7, C18:2n6c, and C18:3n3 descended in both WEE- and FEE-supplemented groups compared with the blank control, whereas the contents of C18:0 and C18:1n9c were elevated in these two groups in comparison with the blank control. These changes in nutritional components showed that feeding inclusion of WEE and FEE shared similar profiles regarding egg nutrient changes. As a result of these changes, the flavor of eggs would also change. Additionally, the fingerprint of the volatile compounds in eggs was established by us via a combination of HS-GC-IMS detection and PLS-DA analysis together and further certified that the dietary supplementation of *E. ulmoides* extracts affected the flavor of eggs, exhibiting significant alteration in volatile egg components [11]. Consistently, the volatile components of eggs in the blank control and the WEE/FEE-supplemented groups were clearly separated in PCA and OPLS-DA analysis, wherein the profiles of volatile components in the WEE-supplemented group were closer to the blank control when compared with the FEE-supplemented group. Therefore, dietary supplementation with FEE created alterations in the eggs’ volatile components. Regardless of many more alterations in volatile egg components being found, the similar profiles of egg nutrients in the WEE- and FEE-supplemented groups suggested that these changes might share the same metabolic process via gut microbial mediation. In the present study, the keystone species *Subdoligranulum* exhibited significant positive correlations with amino acid metabolism, and the other keystone species (*Frankiales*, *Romboutsia*, and UCG-005) were tightly involved in histine (His) decreases. These results certified that dietary supplementation with FEE/WEE could change the composition of bitter amino acids, which was in accordance with our former reports, wherein the dietary supplementation of *E. ulmoides* extract was conducive to bitter amino acid alteration [11]. Meanwhile, four keystone species (V36, V76, V23, and V34) were associated strongly with fatty acid (C18:0 and C18:1n9c) elevation with the dietary addition of FEE/WEE. Hence, it is clear that the feeding inclusion of FEE/WEE caused egg nutrition component alteration that was mediated by gut microbial variation, which was exhibited in a Sankey plot. Based on these points, we therefore affirm that the inclusion of FEE in feed contributes to egg flavor alteration via gut microbial mediation.

## 5. Conclusions

In the present study, a fermented *E. ulmoides* leaf extract (FEE) was prepared and applied in the hen industry after a safety validation. Firstly, the dietary supplementation of FEE did not induce oxidative injury, inflammatory reactions, or histopathological lesions in rats, suggesting that FEE is suitable for long-term feeding. Secondly, an improvement in laying performance, with the egg quality remaining normal, was found with daily supplementation of FEE as compared with daily supplementation of WEE, indicating that daily supplementation of FEE promoted egg production. Thirdly, variations in albumen amino acid, yolk fatty acid, and egg volatile components were found with the dietary addition of FEE, implying that dietary supplementation of FEE was beneficial in terms of egg flavor changes. Lastly, the improved laying performance and egg flavor alteration were strongly associated with the mediating role of FEE in the gut microbiota, which increased the abundance of probiotics. These results could broaden the application of fermented *E. ulmoides* leaf extracts, which serves as a better feed additive alternative than a water extract, in the hen industry.

## Figures and Tables

**Table 1:** Effects of feeding inclusion fermented and water extracted leaf extracts of *E. ulmoides* on laying and growth performance of hens. Different small letters indicate significant differences at *p* < 0.05 level under different treatments. Abbreviations: A group, control; B group, dietary supplementation water extracted leaf extract of *E. ulmoides*; C group, dietary supplementation fermented leaf extract of *E. ulmoides*; BER, broken egg rate; DFC, daily feed consumption; FCR, feed conversion rate.

	Group	A	B	C
Laying performance	Laying rate (%)	89.23	91.24	93.44
	Egg weight (g)	62.43 ± 2.33	64.44 ± 3.44	62.45 ± 3.80
	BER (%)	0.12 ± 0.01 a	0.08 ± 0.01 b	0.09 ± 0.01 a
	DFC (g)	103.08 ± 10.11	103.66 ± 9.78	106.18 ± 11.10
	FCR	1.82	1.77	1.85
	Initial body weight (kg)	1.15 ± 0.14	1.14 ± 0.20	1.09 ± 0.15
	Final body weight (kg)	1.62 ± 0.11	1.63 ± 0.08	1.61 ± 0.12
Organ coefficient	Liver (%)	2.53 ± 0.44	2.32 ± 0.26	2.28 ± 0.30
	Spleen (%)	0.10 ± 0.01	0.10 ± 0.02	0.10 ± 0.01

**Table 2:** Variations in albumen amino acids as feeding inclusion fermented and water extracted leaf extracts of *E. ulmoides*. Different small letters indicate significant differences at *p* < 0.05 level under different treatments. Abbreviations: A group, control; B group, dietary supplementation water extracted leaf extract of *E. ulmoides*; C group, dietary supplementation fermented leaf extract of *E. ulmoides*; DAA, delicious amino acids; SAA, sweet amino acids; BAA, bitter amino acids; EAA, essential amino acids; TAA, total amino acids.

Item	Amino Acid (g/100 g)	A	B	C
Delicious amino acid (DAA)	Asp	1.38 ± 0.03	1.32 ± 0.04	1.38 ± 0.04
	Glu	1.98 ± 0.03	1.95 ± 0.05	2.02 ± 0.04
	Tyr	0.57 ± 0.01	0.55 ± 0.02	0.59 ± 0.02
	Gly	0.47 ± 0.02	0.45 ± 0.01	0.47 ± 0.01
	Phe	0.76 ± 0.01	0.73 ± 0.02	0.77 ± 0.02
	Ala	0.77 ± 0.02	0.75 ± 0.01	0.79 ± 0.02
Sweet amino acid (SAA)	Lys	1.09 ± 0.01	1.06 ± 0.03	1.09 ± 0.02
	Pro	0.48 ± 0.02	0.46 ± 0.02	0.49 ± 0.03
	Ser	1.03 ± 0.02	1.01 ± 0.02	1.05 ± 0.04
	Thr	0.67 ± 0.01	0.65 ± 0.03	0.68 ± 0.03
Bitter amino acid (BAA)	Val	0.86 ± 0.03	0.83 ± 0.04	0.88 ± 0.03
	Leu	1.15 ± 0.03	1.11 ± 0.04	1.18 ± 0.03
	Met	0.44 ± 0.01	0.42 ± 0.01	0.45 ± 0.02
	Arg	0.88 ± 0.02 b	0.87 ± 0.02 b	0.98 ± 0.01 a
	His	0.44 ± 0.01 a	0.36 ± 0.01 b	0.34 ± 0.01 b
	Ile	0.69 ± 0.02	0.68 ± 0.01	0.71 ± 0.03
EAA/TAA (%)		41.43	41.06	40.74
DAA/TAA (%)		43.41	43.56	43.40

**Table 3:** Variations in yolk fatty acids as feeding inclusion fermented and water extracted leaf extracts of *E. ulmoides*. Different small letters indicate significant differences at *p* < 0.05 level under different treatments. Abbreviations: A group, control; B group, dietary supplementation water extracted leaf extract of *E. ulmoides*; C group, dietary supplementation fermented leaf extract of *E. ulmoides*.

Items		A	B	C
Fatty acids (g/100 g)	C14:0	0.11 ± 0.01	0.11 ± 0.01	0.11 ± 0.02
	C14:1n5	0.03 ± 0.01	0.03 ± 0.01	0.03 ± 0.01
	C15:0	0.02 ± 0.01	0.02 ± 0.01	0.02 ± 0.01
	C16:0	9.03 ± 0.11	9.04 ± 0.16	8.96 ± 0.13
	C16:1n7	1.31 ± 0.04 a	1.19 ± 0.02 b	1.08 ± 0.02 c
	C17:0	0.05 ± 0.01	0.05 ± 0.01	0.06 ± 0.01
	C18:0	2.64 ± 0.13 b	2.95 ± 0.09 a	3.00 ± 0.12 a
	C18:1n9t	0.03 ± 0.01	0.04 ± 0.01	0.04 ± 0.01
	C18:1n9c	13.30 ± 0.15 c	13.80 ± 0.10 b	14.10 ± 0.11 a
	C18:2n6t	0.01 ± 0.01	0.01 ± 0.01	0.01 ± 0.01
	C18:2n6c	5.40 ± 0.21 a	4.79 ± 0.16 b	4.81 ± 0.21 b
	C20:0	0.01 ± 0.01	0.01 ± 0.01	0.01 ± 0.01
	C18:3n6	0.03 ± 0.01	0.03 ± 0.01	0.03 ± 0.01
	C18:3n3	0.25 ± 0.01 a	0.20 ± 0.01 b	0.19 ± 0.01 b
	C20:1n9	0.07 ± 0.01	0.08 ± 0.01	0.07 ± 0.02
	C20:2n6	0.05 ± 0.01	0.05 ± 0.01	0.05 ± 0.01
	C22:0	0.03 ± 0.01	0.03 ± 0.01	0.02 ± 0.01
	C20:3n6	0.06 ± 0.01	0.06 ± 0.01	0.06 ± 0.01
	C20:4n6	0.75 ± 0.08	0.76 ± 0.04	0.78 ± 0.03
	C24:1n9	0.01 ± 0.01	0.01 ± 0.01	0.01 ± 0.01
	C22:6n3	0.27 ± 0.02	0.26 ± 0.01	0.29 ± 0.03
	Total	33.46 ± 0.72	33.52 ± 0.76	33.73 ± 0.69

**Table 4:** Effects on blood biochemical parameters as feeding inclusion fermented and water extracted leaf extracts of *E. ulmoides*. Different small letters indicate significant differences at *p* < 0.05 level under different treatments. Abbreviations: A group, control; B group, dietary supplementation water extracted leaf extract of *E. ulmoides*; C group, dietary supplementation fermented leaf extract of *E. ulmoides*; ALT, alanine aminotransferase; AST, aspartate aminotransferase; ALB, albumin; ALP, alkaliphosphatase; BUN, blood urea nitrogen; UA, uric acid; TNF-*α*, tumor necrosis factor-*α*; IL-1, interleukin-1; IL-6, interleukin-6.

	Item	A	B	C
Liver function	ALT (U/L)	2.03 ± 0.26	2.13 ± 0.39	1.70 ± 0.50
	AST (U/L)	192.12 ± 19.09	220.82 ± 54.69	207.72 ± 16.77
	ALB (g/L)	18.00 ± 1.00	18.05 ± 0.98	17.32 ± 2.29
	ALP (U/L)	401.82 ± 12.54 a	374.02 ± 15.11 b	357.77 ± 12.18 b
Nitrogen metabolism	UREA (umol/L)	4.74 ± 0.67 a	4.17 ± 0.37 a	3.33 ± 0.53 b
	BUN (mmol/L)	0.43 ± 0.02	0.42 ± 0.01	0.41 ± 0.02
	UA (umol/L)	232.33 ± 13.23 a	189.61 ± 14.62 b	192.38 ± 17.51 b
Shell-strength-related element	Ca (mmol/L)	9.77 ± 0.65 a	7.73 ± 1.20 b	7.06 ± 1.64 b
	P (mmol/L)	2.00 ± 0.20 a	1.87 ± 0.17 ab	1.55 ± 0.24 b
Inflammatory factory	TNF-*α* (ng/L)	46.48 ± 3.82	44.65 ± 4.61	48.44 ± 2.29
	IL-1 (ng/L)	238.66 ± 16.70	222.95 ± 19.93	219.21 ± 17.58
	IL-6 (ng/L)	32.38 ± 1.54	31.24 ± 1.59	33.34 ± 1.79

## Data Availability

The original contributions presented in the study are included in the article, further inquiries can be directed to the corresponding authors.

## Supplementary Materials

The following supporting information can be downloaded at: https://www.mdpi.com/article/10.3390/foods13101521/s1, Figure S1: Effect of feeding inclusion fermented leaf extract of *E. ulmoides* on the selected tissues of rats (200×); Table S1: The active components between fermented and water extracted leaf extracts of *E. ulmoides*; Table S2: The composition and nutrient levels of basal diet for hens; Table S3: Effects of feeding inclusion fermented leaf extract of *E. ulmoides* on the growth of rats; Table S4: Effects of feeding inclusion fermented leaf extract of *E. ulmoides* on blood routine examination of rats; Table S5: Effects of feeding inclusion fermented leaf extract of *E. ulmoides* on blood biochemistry of rats; Table S6: The antioxidant and inflammatory levels of rats as feeding inclusion fermented leaf extract of *E. ulmoides*; Table S7: The egg quality related indexes among the three tested groups; Table S8: The detected volatile components in yolks; Table S9: Safety assessment of eggs.
