# Supplementary material for: Comparative Analysis of the Effect of Dietary Supplementation with Fermented and Water-Extracted Leaf Extracts of Eucommia ulmoides on Egg Production and Egg Nutrition
Source: Foods. 2024 May 14;13(10):1521. doi: 10.3390/foods13101521 (PMC11120420; doi:10.3390/foods13101521)
Supplement: Supplementary file 1 [file foods-13-01521-s001.zip › foods-2952775-supplementary.pdf]

Supplementary Material

Comparative analysis the effect of dietary supplementation fermented and water extracted *Eucommia ulmoides* leaf extracts on egg production and egg nutrition

Juanhua Yang [a,#], Yunfan Wang [a,#], Lingyan Zheng [a], Mijun Peng [a], Yongzhai Mai [b,*], and Xuesong Wang [a,*]

[a] Chinese Academy of Inspection & Quarantine Greater Bay Area, Zhongshan 528437, China;

[b] Scientific Observing and Experimental Station of Fishery Resources and Environment in the Middle and Lower Reaches of Pearl River, Key Laboratory of Prevention and Control for Aquatic Invasive Alien Species, Fishery Ecological Environment Monitoring Center of Pearl River Basin, Ministry of Agriculture and Rural Affairs, Guangdong Provincial Key Laboratory of Aquatic Animal Immunology and Sustainable Aquaculture, Pearl River Fisheries Research Institute, Chinese Academy of Fishery Sciences, Guangzhou 510380, China;

[#] These authors (J. Yang and Y. Wang) contribute equally to this work.

Corresponding Author:

Yongzhai Mai Email: yongzhanmai@prfri.ac.cn;

Xuesong Wang Email: wangxs@caiqgba.org.cn;

Tel: +86 2084113220

Fax number: +86 2084113220

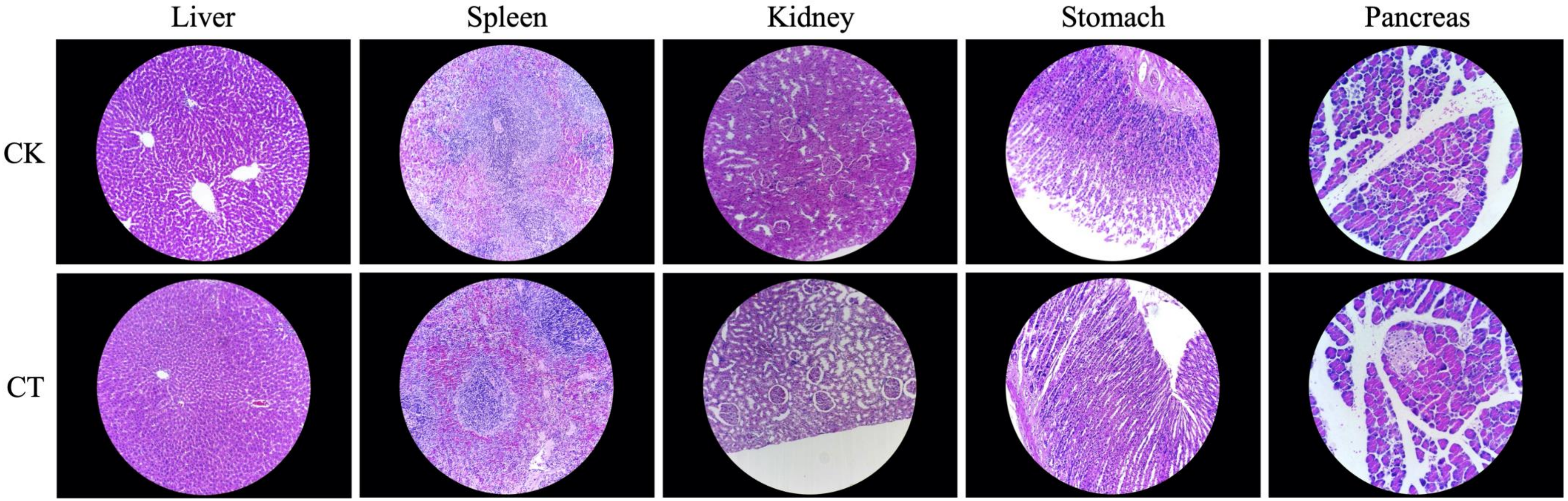

Fig. S1. Effect of feeding inclusion fermented leaf extract of *E. ulmoides* on the selected tissues of rats (200x).

Table S1. The active components between fermented and water extracted leaf extracts of *E. ulmoides*. Abbreviations: WEE, water extracted leaf extract of *E. ulmoides*; FEE, fermented leaf extract of *E. ulmoides*.

Index	WEE	FEE
Total polyphenol (g/100g)	17.35 ± 0.61 a	12.78 ± 0.44 b
Total flavonoid (g/100g)	5.27 ± 0.06 a	3.44 ± 0.29 b
Chlorogenic acid (mg/g)	81.73 ± 3.73 a	54.33 ± 3.73 b
Aucubin (mg/g)	55.51 ± 0.43 a	30.48 ± 0.31 b
Geniposidic acid (mg/g)	86.55 ± 0.56 a	62.77 ± 0.49 b
Rutin (mg/g)	7.55 ± 0.18 a	4.98 ± 0.28 b

Table S2. The composition and nutrient levels of basal diet for hens.

Ingredient	Composition
Corn	62.73
Soybean meal	20.58
Limestone	5
Dicalcium phosphate	8.96
Soybean oil	1.27
Vitamin premix [a]	0.6
Minerals premix [b]	0.02
Choline chloride	0.1
Salt	0.3
L-Methionine	0.34
Total	100
Calculated chemical composition (%)	
ME (kcal/kg)	2658
Crude protein	17
Calcium	3.49
Total phosphorus	0.55
Lysine	0.8
Methione	0.379

[a] Vitamin premix provide the following per kilogram of diet: vitamin A, 12,500 IU; vitamin D3, 2,500 IU; vitamin E, 15 IU; vitamin K3, 2.65 mg; vitamin B1, 2 mg; vitamin B2, 6 mg; vitamin B12, 0.025 mg; nicotinic acid, 50 mg; calcium pantothenate, 12 mg; biotin, 0.0325 mg; folic acid, 1.25 mg.

[b] The mineral premix provide the following per kg of diet: iron, 80 mg; copper, 8 mg; manganese, 100 mg; zinc, 75 mg; iodine, 0.35 mg; selenium, 0.15 mg.

Table S3. Effects of feeding inclusion fermented leaf extract of *E. ulmoides* on the growth of rats. Different small letters indicate significant differences at $P < 0.05$ level under different treatments. Abbreviations: CK group, control; CT group, dietary supplementation fermented leaf extract of *E. ulmoides*.

Sex	Male		Female	
Group	CK	CT	CK	CT
Body weight (g)	334.21 ± 14.94 b	377.45 ± 25.52 a	243.8 ± 16.10	250.6 ± 16.78
Brain (g)	1.49 ± 0.02 b	1.79 ± 0.10 a	1.77 ± 0.11	1.79 ± 0.16
Liver (g)	7.91 ± 0.52	8.32 ± 0.57	6.59 ± 0.47	6.09 ± 0.49
Spleen (g)	0.54 ± 0.07	0.62 ± 0.09	0.50 ± 0.05	0.52 ± 0.06
Heart (g)	0.89 ± 0.04 b	1.01 ± 0.08 a	0.78 ± 0.02	0.72 ± 0.06
Kidney (g)	1.87 ± 0.13 b	2.12 ± 0.09 a	1.52 ± 0.10	1.58 ± 0.13
Testis (g)	3.52 ± 0.04	3.53 ± 0.02	\	\

Table S4. Effects of feeding inclusion fermented leaf extract of *E. ulmoides* on blood routine examination of rats. Different small letters indicate significant differences at $P < 0.05$ level under different treatments. Abbreviations: CK group, control; CT group, dietary supplementation fermented leaf extract of *E. ulmoides*; WBC, white blood cell; Neu, neutrophil; Lym, lymphocyte; Mon, monocyte; Eos, eosinophil; Bas, basophil; RBC, red blood cell; HGB, hemoglobin; HCT, hematocrit value; MCV, mean corpuscular volume; MCH, mean corpuscular hemoglobin; MCHC, mean corpusular hemoglobin concerntration; RDW-CV, red blood cell distribution width-coefficient of variation; RDW-SD, red blood cell distribution width-standard deviation; PLT, platelet; MPV, mean platelet volume; PDW, platelet distribution width; PCT, thrombocytocrit.

Sex	Index	CK	CT
Male	WBC (10^9/L)	4.88 ± 1.48	5.37 ± 1.31
	Neu (10^9/L)	0.67 ± 0.17	0.53 ± 0.12
	Lym (10^9/L)	3.69 ± 1.49	4.65 ± 1.15
	Mon (10^9/L)	0.26 ± 0.11	0.22 ± 0.08
	Eos (10^9/L)	0.03 ± 0.02	0.01 ± 0.02
	Bas (10^9/L)	0.01 ± 0.01	0.01 ± 0.01
	Neu (%)	16.01 ± 5.54	9.74 ± 1.08
	Lym (%)	77.41 ± 5.99	86.03 ± 1.59
	Mon (%)	5.59 ± 0.62	3.98 ± 0.91

	Eos (%)	0.84 ± 0.55	0.19 ± 0.22
	Bas (%)	0.19 ± 0.11	0.07 ± 0.07
	RBC (10^{12}/L)	9.21 ± 0.55	9.14 ± 0.42
	HGB (g/L)	163.13 ± 7.36	164.12 ± 2.93
	HCT (%)	47.11 ± 2.28	46.25 ± 1.91
	MCV (fL)	51.24 ± 0.99	51.13 ± 0.98
	MCH (pg)	17.79 ± 0.49	18.26 ± 0.52
	MCHC (g/L)	349.92 ± 5.32	359.36 ± 6.53
	RDW-CV (%)	12.77 ± 0.28	13.09 ± 0.34
	RDW-SD (fL)	26.38 ± 0.42	27.43 ± 0.68
	PLT (10^9/L)	902.81 ± 118.26	968.48 ± 97.65
	MPV (fL)	6.25 ± 0.32	6.44 ± 0.26
	PDW	15.29 ± 0.14	15.19 ± 0.11
	PCT (%)	0.56 ± 0.09	0.62 ± 0.07
Female	WBC (10^9/L)	4.73 ± 1.19	3.95 ± 1.82
	Neu (10^9/L)	0.55 ± 0.16	0.62 ± 0.23
	Lym (10^9/L)	3.86 ± 1.02	3.18 ± 1.51
	Mon (10^9/L)	0.28 ± 0.07	0.25 ± 0.13
	Eos (10^9/L)	0.04 ± 0.02	0.02 ± 0.01
	Bas (10^9/L)	0.01 ± 0.01	0.01 ± 0.01
	Neu (%)	11.52 ± 1.94	13.71 ± 3.31
	Lym (%)	81.28 ± 2.63	80.03 ± 4.41

	Mon (%)	6.07 ± 1.64	6.32 ± 1.86
	Eos (%)	0.81 ± 0.41	0.52 ± 0.28
	Bas (%)	0.34 ± 0.13	0.11 ± 0.09
	RBC (10^{12}/L)	8.46 ± 0.33	8.81 ± 0.56
	HGB (g/L)	157.94 ± 5.76	161.44 ± 6.08
	HCT (%)	44.46 ± 1.68	45.75 ± 2.49
	MCV (fL)	52.55 ± 0.91	52.24 ± 1.18
	MCH (pg)	18.67 ± 0.33	18.59 ± 0.56
	MCHC (g/L)	355.44 ± 3.78	358.28 ± 6.78
	RDW-CV (%)	12.13 ± 0.39	12.47 ± 0.41
	RDW-SD (fL)	26.25 ± 0.74	27.12 ± 0.83
	PLT (10^{9}/L)	1080.92 ± 143.44	1181.91 ± 142.35
	MPV (fL)	6.37 ± 0.17	6.21 ± 0.32
	PDW	15.27 ± 0.18	15.13 ± 0.17
	PCT (%)	0.67 ± 0.08	0.70 ± 0.06

Table S5. Effects of feeding inclusion fermented leaf extract of *E. ulmoides* on blood biochemistry of rats. Different small letters indicate significant differences at $P < 0.05$ level under different treatments. Abbreviations: CK group, control; CT group, dietary supplementation fermented leaf extract of *E. ulmoides*; ALB, albumin; ALP, alkaliphosphatase; ALT, alanine aminotransferase; AST, aspartate aminotransferase; CREA-S, creatinine; Glu-G, glucose; TC, total cholesterol; TG, triglyceride; TP, total phosphorus; γ-GT, γ-glutamyl transpeptidase.

Sex	Index	CK	CT
Male	ALB (g/L)	33.37 ± 1.43	33.82 ± 2.93
	ALP (U/L)	113.36 ± 8.09	101.71 ± 24.46
	ALT (U/L)	35.31 ± 5.82	24.51 ± 19.14
	AST (U/L)	88.34 ± 15.43	103.48 ± 19.50
	CREA-S (μmol/L)	41.32 ± 11.34	45.97 ± 7.96
	Glu-G (mmol/L)	9.17 ± 1.03	9.23 ± 1.21
	TC (mmol/L)	2.45 ± 0.28	2.26 ± 0.13
	TG (mmol/L)	0.60 ± 0.13	0.44 ± 0.11
	TP (g/L)	57.20 ± 2.16	58.67 ± 5.06
	UREA (mmol/L)	5.95 ± 2.10	6.51 ± 0.73
	γ-GT (U/L)	2.06 ± 0.93	2.11 ± 0.94
Female	ALB (g/L)	37.82 ± 1.63 a	33.56 ± 2.29 b
	ALP (U/L)	76.31 ± 32.70	77.97 ± 8.85

	ALT (U/L)	25.60 ± 4.22	30.02 ± 6.03
	AST (U/L)	86.86 ± 13.22	116.22 ± 23.36
	CREA-S (μmol/L)	51.15 ± 12.26	54.56 ± 10.89
	Glu-G (mmol/L)	7.23 ± 1.39	6.38 ± 1.41
	TC (mmol/L)	1.96 ± 0.24	2.29 ± 0.50
	TG (mmol/L)	0.82 ± 0.41	0.66 ± 0.20
	TP (g/L)	63.32 ± 2.56	59.97 ± 4.87
	UREA (mmol/L)	7.38 ± 1.43	6.26 ± 0.99
	γ-GT (U/L)	1.03 ± 0.46	1.20 ± 0.38

Table S6. The antioxidant and inflammatory levels of rats as feeding inclusion fermented leaf extract of *E. ulmoides*. Different small letters indicate significant differences at $P < 0.05$ level under different treatments. Abbreviations: CK group, control; CT group, dietary supplementation fermented leaf extract of *E. ulmoides*; SOD, superoxide dismutase; GSH, glutathione; MDA, molondialdehyde; CAT, catalase; TNF-α, tumor necrosis factor-α; IL-1β, interleukin-1β; IL-6, interleukin-6.

Sex	Index		CK	CT
Male	Antioxidant index	SOD (U/ml)	107.31 ± 17.70	110.97 ± 19.92
		GSH (μmol/L)	1.95 ± 0.32	2.02 ± 0.73
		MDA (nmol/L)	8.43 ± 1.56	9.14 ± 1.95
		CAT (U/ml)	15.6 ± 2.57	16.14 ± 2.81
	Inflammatory factor	TNF-α (ng/L)	378.84 ± 36.92	383.03 ± 25.09
		IL-1β (ng/L)	42.04 ± 3.85	43.81 ± 3.48
		IL-6 (pg/ml)	122.95 ± 10.75	120.51 ± 5.39
Female	Antioxidant index	SOD (U/ml)	110.83 ± 20.62	114.19 ± 15.14
		GSH (μmol/L)	2.74 ± 0.74	2.08 ± 0.82
		MDA (nmol/L)	9.19 ± 1.56	9.69 ± 1.93
		CAT (U/ml)	16.94 ± 5.91	16.61 ± 6.57
	Inflammatory factor	TNF-α (ng/L)	385.96 ± 26.09	388.96 ± 26.56
		IL-1β (ng/L)	41.95 ± 3.24	43.03 ± 3.13
		IL-6 (pg/ml)	119.73 ± 10.61	120.69 ± 9.21

Table S7. The egg quality related indexes among the three tested groups. Different small letters indicated significant differences at *P* < 0.05 level under different treatments. Abbreviations: A group, control; B group, dietary supplementation water extracted leaf extract of *E. ulmoides*; C group, dietary supplementation fermented leaf extract of *E. ulmoides*.

Items	A	B	C
Yolk color	6.60 ± 0.55	6.56 ± 0.35	6.48 ± 0.45
Egg-shape index	1.30 ± 0.02	1.28 ± 0.02	1.31 ± 0.01
Shell strength (N)	38.79 ± 7.29	38.60 ± 7.27	38.57 ± 6.72
Shell weight (g)	5.71 ± 0.33	5.79 ± 0.49	6.08 ± 0.51
Shell thickness (mm)	0.32 ± 0.01	0.32 ± 0.01	0.33 ± 0. 01
Egg albumen height (mm)	8.72 ± 0.87	8.10 ± 0.70	7.90 ± 0.70
Haugh unit	92.50 ± 4.10	88.92 ± 3.37	87.74 ± 4.12
Percentage of yolk (%)	25.86 ± 1.52	26.20 ± 1.22	26.36 ±0.94
Yolk moisture content (%)	48.81 ± 0.33	48.90 ± 0.29	48.16 ± 0.48
Albumen moisture content (%)	88.25 ± 0.32	88.32 ± 0.43	87.93 ± 0.46

Table S8. The detected volatile components in yolks. MW, molecular weight; RI, retention index; RT, retention time; DT, migration time; RIP Rel, normalized treatment.

Count	Compound	CAS#	Formula	MW	RI	RT [sec]	DT [RIP Rel]	Comment
1	Benzaldehyde	C100527	C7H6O	106.1	1549.8	1416.033	1.16375	
2	Acetic acid	C64197	C2H4O2	60.1	1504.4	1283.096	1.05957	monomer
3	Acetic acid	C64197	C2H4O2	60.1	1503.7	1281.169	1.151	dimer
4	1-Octen-3-ol	C3391864	C8H16O	128.2	1480.9	1219.517	1.17438	
5	Methional	C3268493	C4H8OS	104.2	1473.6	1200.251	1.10209	
6	(E)-2-Octenal	C2548870	C8H14O	126.2	1437.5	1109.7	1.34236	
7	Ethyl octanoate	C106321	C10H20O2	172.3	1432.6	1098.141	1.46355	
8	(Z)-Hex-3-enol	C928961	C6H12O	100.2	1417.0	1061.535	1.26156	
9	Nonanal	C124196	C9H18O	142.2	1401.7	1026.856	1.48269	
10	1-Hexanol	C111273	C6H14O	102.2	1368.4	955.202	1.3342	
11	Ethyl heptanoate	C106309	C9H18O2	158.2	1340.6	899.412	1.39373	

12	(E)-2-Heptenal	C18829555	C7H12O	112.2	1332.8	884.302	1.26018	monomer
13	(E)-2-Heptenal	C18829555	C7H12O	112.2	1332.2	883.14	1.66888	dimer
14	2,5-Dimethylpyrazine	C123320	C6H8N2	108.1	1332.2	883.14	1.09767	
15	1-Hydroxy-2-propanone	C116096	C3H6O2	74.1	1314.3	849.433	1.04618	
16	1-Octen-3-one	C4312996	C8H14O	126.2	1310.5	842.46	1.26823	
17	Octanal	C124130	C8H16O	128.2	1298.3	820.376	1.40338	
18	4-Methyl-1-pentanol	C626891	C6H14O	102.2	1296.9	818.052	1.64796	
19	Cyclohexanone	C108941	C6H10O	98.1	1295.4	815.372	1.16098	
20	1-Pentanol	C71410	C5H12O	88.1	1264.8	765.254	1.25585	monomer
21	1-Pentanol	C71410	C5H12O	88.1	1264.8	765.254	1.50884	dimer
22	2-Methylpyrazine	C109080	C5H6N2	94.1	1275.9	783.004	1.09378	
23	Acetoin	C513860	C4H8O2	88.1	1268.8	771.518	1.0503	

24	2-Pentylfuran	C3777693	C9H14O	138.2	1241.2	728.709	1.25981	
25	(E)-2-Hexenal	C6728263	C6H10O	98.1	1232.1	715.136	1.1847	
26	3-Methyl-1-butanol	C123513	C5H12O	88.1	1220.7	698.43	1.24795	
27	Cyclopentanone	C120923	C5H8O	84.1	1214.9	690.077	1.09773	
28	Heptanal	C111717	C7H14O	114.2	1197.8	666.062	1.33096	monomer
29	Heptanal	C111717	C7H14O	114.2	1197.0	665.018	1.70056	dimer
30	2-Heptanone	C110430	C7H14O	114.2	1192.5	658.753	1.26771	
31	Isoamyl isovalerate	C659701	C10H20O2	172.3	1299.0	821.636	1.46733	
32	1-Penten-3-ol	C616251	C5H10O	86.1	1177.1	626.385	0.94752	
33	Ethyl crotonate	C623701	C6H10O2	114.1	1177.6	627.429	1.58592	
34	1-Butanol	C71363	C4H10O	74.1	1161.6	595.062	1.18668	monomer
35	1-Butanol	C71363	C4H10O	74.1	1161.6	595.062	1.37642	dimer
36	(E)-2-Pentenal	C1576870	C5H8O	84.1	1149.2	571.047	1.10959	monomer

37	(E)-2-Pentenal	C1576870	C5H8O	84.1	1150.3	573.135	1.36456	dimer
38	p-Xylene	C106423	C8H10	106.2	1146.5	565.826	1.07797	
39	2-Butylfuran	C4466244	C8H12O	124.2	1135.7	545.988	1.20842	
40	Hexanal	C66251	C6H12O	100.2	1100.3	485.429	1.26178	monomer
41	Hexanal	C66251	C6H12O	100.2	1100.9	486.473	1.56616	dimer
42	4-Methyl-3-penten-2-one	C141797	C6H10O	98.1	1126.9	530.326	1.1175	
43	2-Methyl-1-propanol	C78831	C4H10O	74.1	1109.8	501.09	1.17482	
44	1-Propanol	C71238	C3H8O	60.1	1054.5	423.276	1.11542	monomer
45	1-Propanol	C71238	C3H8O	60.1	1056.0	425.224	1.24991	dimer
46	Thiophene	C110021	C4H4S	84.1	1029.5	393.087	1.0297	
47	1-Penten-3-one	C1629589	C5H8O	84.1	1041.8	407.695	1.07995	
48	2-Butanol	C78922	C4H10O	74.1	1041.8	407.695	1.14645	monomer

49	2-Butanol	C78922	C4H10O	74.1	1041.0	406.721	1.31789	dimer
50	Isobutyl acetate	C110190	C6H12O2	116.2	1027.0	390.166	1.23513	
51	Propyl acetate	C109604	C5H10O2	102.1	1026.1	389.192	1.48194	dimer
52	Propyl acetate	C109604	C5H10O2	102.1	1026.1	389.192	1.1568	monomer
53	Pentanal	C110623	C5H10O	86.1	998.9	359.003	1.17749	monomer
54	Pentanal	C110623	C5H10O	86.1	998.9	359.003	1.42578	dimer
55	2-Pentanone	C107879	C5H10O	86.1	998.9	359.003	1.36961	
56	Ethanol	C64175	C2H6O	46.1	950.2	321.024	1.04595	monomer
57	Ethanol	C64175	C2H6O	46.1	950.2	321.024	1.1302	dimer
58	2-Methylbutanal	C96173	C5H10O	86.1	921.0	300.573	1.17601	
59	3-Methylbutanal	C590863	C5H10O	86.1	926.7	304.468	1.41395	
60	tert-Butanol	C75650	C4H10O	74.1	928.1	305.442	1.33267	
61	2-Butanone	C78933	C4H8O	72.1	915.2	296.678	1.24991	

62	Ethyl Acetate	C141786	C4H8O2	88.1	892.8	282.07	1.34153
63	Diethyl sulfide	C352932	C4H10S	90.2	877.2	272.332	1.21148
64	Acrolein	C107028	C3H4O	56.1	864.3	264.541	1.0829
65	Acetone	C67641	C3H6O	58.1	839.1	249.934	1.12281
66	Propanal	C123386	C3H6O	58.1	806.8	232.404	1.14645
67	Ethyl formate	C109944	C3H6O2	74.1	819.6	239.221	1.21739
68	2-Methylpropanal	C78842	C4H8O	72.1	819.6	239.221	1.28537
69	Dimethyl sulphide	C75183	C2H6S	62.1	795.5	226.561	0.95728
70	Acetaldehyde	C75070	C2H4O	44.1	757.7	208.059	0.98241
71	Diethyl acetal	C105577	C6H14O2	118.2	906.4	290.835	1.03265
72	Myrcene	C123353	C10H16	136.2	1176.4	624.86	1.21887
73	Dimethylamine	C124403	C2H7N	45.1	1226.4	706.662	0.84792
74	Trimethylamine	C75503	C3H9N	59.1	1227.0	707.636	0.89521

Table S9. Safety assessment of eggs. Abbreviations: A group, control; B group, dietary supplementation water extracted leaf extract of *E. ulmoides*; C group, dietary supplementation fermented leaf extract of *E. ulmoides*.

Item	Index	A	B	C
Microorganisms	*Salmonella* (CFU/mL)	-	-	-
	Aerobic plate count (CFU/mL)	< 100	< 100	< 100
	Moulds (CFU/mL)	< 3	< 3	< 3
	Coliforms (CFU/mL)	< 10	< 10	< 10
Aflatoxins	Aflatoxin B1 (μ/kg)	-	-	-
Heavy metal	Pb (mg/kg)	-	-	-
	Cd (mg/kg)	-	-	-
